# Fungal Diversity in the Neptune Forest: Comparison of the Mycobiota of *Posidonia oceanica*, *Flabellia petiolata*, and *Padina pavonica*

**DOI:** 10.3389/fmicb.2020.00933

**Published:** 2020-05-26

**Authors:** Anna Poli, Elena Bovio, Lucrezia Ranieri, Giovanna Cristina Varese, Valeria Prigione

**Affiliations:** Department of Life Sciences and Systems Biology, Mycotheca Universitatis Taurinensis, University of Torino, Turin, Italy

**Keywords:** marine fungi, Mediterranean Sea, seagrass, algae, phylogeny, Lulworthiales

## Abstract

Fungi are widely distributed in the Oceans, interact with other organisms and play roles that range from pathogenic to mutualistic. The present work focuses on the characterization of the cultivable mycobiota associated with the seagrass *Posidonia oceanica* (L.) Delile collected off the Elba Island (Italy). We identified 102 taxa (mainly Ascomycota) by the mean of a polyphasic approach. Leaves, rhizomes, roots and matte were characterized by unique mycobiota revealing a “plant-part-specificity.” The comparison with the mycobiota associated with the green alga *Flabellia petiolata* and the brown alga *Padina pavonica* underlined a “substrate specificity.” Indeed, despite being part of the same phytocoenosis, these photosynthetic organisms recruit different fungal communities. The mycobiota seems to be necessary for the host’s defense and protection, playing, in this way, remarkable ecological roles. Among the 61 species detected in association with *P. oceanica* (including two species belonging to the newly introduced genus *Paralulworthia*), 37 were reported for the first time from the Mediterranean Sea.

## Introduction

*Posidonia oceanica* (L.) Delile, otherwise known as Neptune grass, is the most important seagrass in the Mediterranean Sea (Personnic et al., 2014). This endemic long-lived plant (Magnoliophyta, kingdom Archaeplastida) relies mainly on vegetative reproduction, and its largest clones can spread over 15 km (Arnaud-Haond et al., 2012). Neptune grass meadows, or rather forests, are a spawning ground, a nursery and a permanent habitat for many species: the presence of over 400 different seaweeds species and several thousands of animal species makes this underwater habitat a unique biodiversity hotspot (Boudouresque, 2004).

Rhizomes, roots and senescent leaf sheaths of *P. oceanica* form a characteristic peat-like seabed layer (matte) that can be several meters thick and thousands of years old. Matte are extremely resistant to microbiological decay, however, herbivores can feed on them with the aid of symbiotic microorganisms such as fungi or bacteria capable of degrading organic matters (Vohnik et al., 2017). To this respect, it is important to underline that likewise other seagrasses, *P. oceanica* supports a complex ecosystem that supplies large amounts of nutrients to the organisms living within and in adjacent waters. Fungi play an important role in the functioning of this ecosystem: while parasites and patho ens can negatively affect the wellness of the meadows, mutualists may be essential for their health (Vohnik et al., 2019). Finally, saprobes transform dead plants into nutritive detritus through their degrading activity (Raghukumar, 2017). Overall, *Posidonia* forests are home to 20–25% of Mediterranean species and represent the climax community of the Mediterranean Sea (Minelli et al., 2008). However, in the aftermath of the anthropic impact, of the presence of exotic species and of the climate change, this climax community is seriously threatened and since 1992 is a priority in Annex I of the Council Directive 92/43/EEC on the conservation of natural habitats and of wild fauna and flora.

Marine fungi are a huge part of the microbial diversity hosted in the sea. Indeed, they have been retrieved worldwide from biotic and abiotic substrates such as algae, sediments, invertebrates, drift wood, etc. (Jones and Pang, 2012; Garzoli et al., 2014, 2015, 2018; Gnavi et al., 2017; Raghukumar, 2017; Bovio et al., 2018). Nevertheless, despite the total fungal diversity has been estimated to range between 10,000 and 12,500 taxa (Jones et al., 2019), only 1281 species have been described to date^1^. These organisms are important primary decomposers and living as mutualists (ecto- and endosymbionts), parasites, pathogens and saprobes, contribute greatly to nutrient cycling and food web (Amend et al., 2019; Grossart et al., 2019).

As part of a thorough investigation aimed to uncover the fungal diversity of the Mediterranean Sea, we focused on the isolation and identification of the cultivable mycobiota associated with *P. oceanica*, collected off the Elba Island (Tuscany). Once defined, the mycobiota was compared to those associated with the green and brown algae *Flabellia petiolata* (Gnavi et al., 2017) and *Padina pavonica* (Garzoli et al., 2018), two important components of the same phytocoenosis, collected during the same sampling and in close proximity. Finally, this paper introduces the novel genus *Paralulworthia* represented by two new species *P. gigaspora* and *P. padinae*.

## Materials and Methods

### Sampling

Plants of *P. oceanica* were collected from two sampling sites in the coastal waters of the Elba Island (Livorno, Italy): (1) Ghiaie (UTM WGS84 42°49′04″N, 10°19′20″E) and (2) Margidore (UTM WGS84 42°45′29″N, 10°18′24″E). Twelve plants (6 per sampling site) with the surrounding fluctuating senescent leaves (matte) were harvested at depth ranging from 5 to 15 m below sea level (bsl). Plants were maintained in sterile dark containers at 4°C and processed within 36 h from sampling. Specific permissions to operate in the protected area of “Le Ghiaie” (Ghiaie site) and in the freely accessible Margidore site, were obtained by the port authority of Portoferraio (Livorno, Italy).

### Fungal Isolation

**FIGURE 1:**
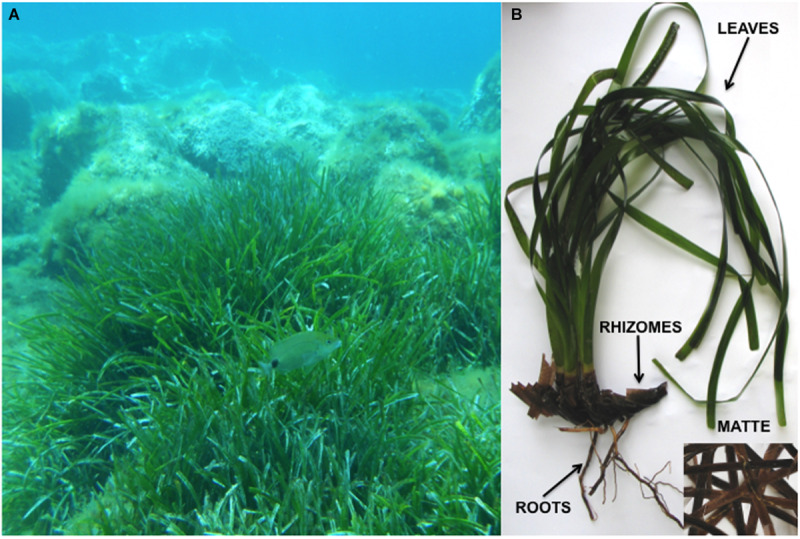
*Posidonia oceanica* medows in its natural habitat **(A)**; a plant of *P. oceanica* with the four plant parts indicated **(B)**.

Each plant was divided into four parts: leaves, rhizomes, roots and matte (Figure 1). In order to remove unrefined sediments, each sample was individually sonicated (30” each time) and serially washed (three times) in sterilized artificial SeaWater (SW, 3.4% w/v Sea Salt mix – Sigma-Aldrich, St. Louis, MO, United States – in distilled water). Individual samples were homogenized in 20 mL of sterile filtered seawater by means of Ultra-Turrax (IKA, Staufen, Germany). One mL of homogenate was plated onto Petri dishes (15 cm Ø) containing the following culture media: Corn Meal Agar SeaWater (CMASW; 17g CMA – Sigma-Aldrich, St. Louis, MO, United States – in 1 L of filtered SW) or Agar Posidonia (AP; 20 g of *P. oceanica* heated in 100 mL seawater – 60°C, 30 min – were filtered prior to the addition of 18 g agar and SW up to 1L). Each medium was supplemented with antibiotics (Gentamicin 80 mg L^–1^, Piperacillin and Tazobactam 100 mg L^–1^ – Sigma-Aldrich, St. Louis, United States).

Three replicates per medium and per sample were performed. A total of 288 plates were incubated at 15°C (spring average temperature of submerged meadows between 5 and 15 m bsl) for 15 days. Afterward, the temperatures was raised to 25°C for 45 days. This procedure allowed the isolation of both psychrophilic and mesophilic fungi. Colony forming units per gram of dry weight of each plant part (CFU g^–1^ dw) were recorded. Isolated fungi were maintained in pure culture.

### Fungal Identification

Fungi were identified by combining morpho-physiological, molecular and phylogenetic studies. Following preliminary determination of genera according to macroscopic and microscopic features (Domsch et al., 1980; von Arx, 1981; Kiffer and Morelet, 1997), fungal isolates were transferred to the media recommended by the authors of selected genus monographs for species identification. Molecular identification was performed by amplifying and sequencing the appropriate genetic marker as previously described (Poli et al., 2016; Garzoli et al., 2018). PCR products were purified and sequenced at Macrogen Europe Laboratory (Madrid, Spain). The resulting ABI chromatograms were visually inspected, trimmed and assembled to obtain consensus sequences using Sequencer 5.0 (GeneCodes Corporation, Ann Arbor, MI, United States)^2^. The newly generated sequences were compared by BLASTn analyses (default settings) to those available in public nucleotide databases provided by the NCBI (Bethesda MD, United States) and by the Westerdijk Fungal Biodiversity Institute (Utrecht, The Netherlands). Similarity values equal or higher than 98% (*e*-value > e-100) were considered credible.

Representative strains of each species isolated in pure culture during this work are preserved at *Mycotheca Universitatis Taurinensis* (MUT)^3^. The accession numbers of the sequences deposited in GenBank are: MK578237 – MK578251; MK581058 – MK581072; MK626711 – MK626722; MN165499 – MN165504; MN173027 – MN173033; MN177616 – MN177627; MN173595 – MN173610.

### *Paralulworthia gigaspora* and *Paralulworthia posidoniae*: Growth Condition, Morphological, and Molecular Study

Following pre-growth, MUT 435, MUT 672, MUT 5413, and MUT 5261 were inoculated in triplicate onto Petri dishes (9 cm Ø) containing MEASW and incubated at 21°C. The fungal growth, as well as macro- and microscopic features, was monitored at 4, 7, 14, 21 and 28 days from the inoculum. In an attempt to induce sporulation, sterile specimens of *Quercus ruber* cork and *Pinus pinaster* wood (species autochthonous to the Mediterranean area) were placed on 3 weeks old fungal colonies (Panebianco et al., 2002) which were further incubated for 4 weeks at 21°C. Colonized wood specimens were transferred into 50 mL tubes containing 20 mL sterile sea water and incubated at 21°C for 1 month. Mature reproductive fungal structures were observed and images captured with an optical microscope (Leica DM4500B, Leica Microsystems GmbH, Germany) equipped with a camera (Leica DFC320, Leica Microsystems GmbH, Germany). For a better inspection of ascomata growing inside wood tissues, scraps of colonized wood embedded in melted paraffin were dissected with a cryostat (Leica CM1850, Leica Microsystems GmbH, Germany).

Genomic DNA was extracted as mentioned above and the partial sequences of the internal transcribed spacers including the 5.8S rDNA gene (*nrITS*), 28S large ribosomal subunit (*nrLSU*) and 18S small ribosomal subunit (*nrSSU*) were amplified by PCR using the primer pairs ITS1/ITS4 (White et al., 1990), LR0R/LR7 (Vilgalys and Hester, 1990), NS1/NS4 (White et al., 1990). A dataset consisting of *nrSSU*, *nrITS*, and *nrLSU*, was assembled on the basis of BLASTn results and of the most recent phylogenetic study focused on Lulworthiales (Azevedo et al., 2017). Reference sequences were retrieved from GenBank (Table 2). Alignments were generated using MUSCLE (default conditions for gap openings and gap extension penalties), implemented in MEGA v. 7.0 (Molecular Evolutionary Genetics Analysis), visually inspected and trimmed by TrimAl v. 1.2^4^ to delimit and discard ambiguously aligned regions. No incongruence was observed among single-loci phylogenetic trees and alignments were concatenated into a single data matrix with SequenceMatrix (Vaidya et al., 2011). The best evolutionary model under the Akaike Information Criterion (AIC) was determined with jModelTest 2 (Darriba et al., 2012). Phylogenetic inference was estimated using Maximum Likehood (ML) and Bayesian Inference (BI) criteria. The ML analysis was produced using RAxML v. 8.1.2 (Stamatakis, 2014) under GTR + I + G evolutionary model and 1,000 bootstrap replicates. BI was performed with MrBayes 3.2.2 (Ronquist et al., 2012) with the same substitution model (GTR + I + G). The alignment was run for 5 million generations with two independent runs each containing four Markov Chains Monte Carlo (MCMC) and sampling every 100 iterations. The first 25% of generated trees were discarded as “burn-in.” A consensus tree was generated using the “sumt” function of MrBayes and Bayesian posterior probabilities (BYPP) were calculated. Consensus trees were visualized in FigTree v. 1.4.2^5^. Due to topological similarity of the two resulting trees, only BI analysis with BYPP and MLB values was reported.

### Statistical Analysis

Differences among the total fungal loads (CFU g^–1^ dwt) of each plant portion were inferred by applying the analysis of variance (ANOVA), Bonferroni *post hoc* test (*p* < 0.05), using GraphPad Prism 5 for Windows, GraphPad Software (San Diego, CA, United States)^6^.

Significant differences among mycobiota of individual plant parts and organisms were evaluated by applying the PERmutational Multivariate ANalysis Of Variance (PERMANOVA; pseudo-F index; *p* < 0.05) and visualized by the Canonical Analysis of Principal Coordinates (CAP) or Principal Coordinate Analysis (PCO). The contribution of single species (in percentage) to the diversity observed within and between groups was assessed by SIMilarity PERcentage (SIMPER) analysis. These analyses were performed with the statistical package PRIMER 7 (Plymouth Routines in Multivariate Ecological Research, Albany Auckland, New Zealand).

## Results

**FIGURE 2:**
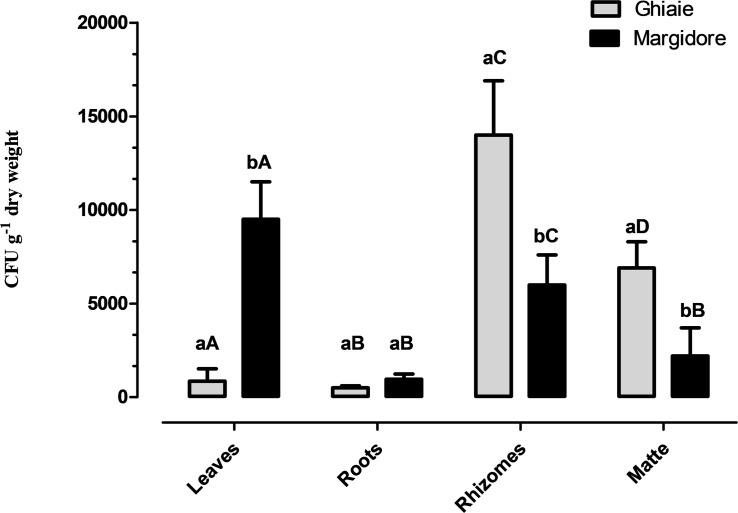
Fungal total load (CFU g^– 1^ dwt) detected in the four parts of *P. oceanica* (leaves, roots, rhizomes, and matte) in Ghiaie and Margidore. Results are expressed as mean ± SD and analyzed through two-way analysis of variance (ANOVA), Bonferroni *post hoc* (*p* < 0.05). Capital letters indicate significant differences within the same sampling site; lowercase letters indicate significant differences between sampling sites.

All plants of *P. oceanica* were colonized by fungi. The average fungal abundance (CFU g^–1^dw) of the four parts ranged from 5.0 × 10^2^ CFU g^–1^dw to 1.4 × 10^4^ CFU g^–1^dw (Figure 2). In Ghiaie, rhizomes displayed the highest fungal load, followed by matte, leaves and roots, whereas in Margidore leaves were the richest part, followed by rhizomes and matte evenly, and finally by roots. Considering the individual plant parts, only roots did not differ significantly between the two sampling sites.

Overall, 287 isolates, representative of 102 taxa were retrieved: 43 from leaves, 70 from matte, 118 from rhizomes, and 56 from roots. The majority of taxa needed a specific medium and incubation temperature: 50 taxa were exclusively isolated from CMASW, 31 from AP and only 21 from both media; 76 taxa grew exclusively at 25°C and 11 at 15°C. In comparison to the plants sampled at Ghiaie, the number of taxa retrieved from the plants harvested at Margidore was almost double (Table 1).

**TABLE 1:** Number of exclusive fungal taxa per site (Ghiaie and Margidore), medium (CMA and AP), temperature (15°C and 25°C) and plant part (leaves, roots, rhizomes, and matte).

		**Ghiaie**	**Margidore**
	Total	27 (53)	49 (75)
Medium	CMA	23 (43)	35 (53)
	AP	10 (29)	22 (40)
Temperature	15°C	3 (15)	9 (23)
	25°C	38 (50)	52 (66)
Plant part	Leaves	6 (20)	9 (21)
	Roots	4 (14)	9 (18)
	Rhizomes	14 (31)	22 (38)
	Matte	4 (24)	11 (31)

*In brackets total number of taxa retrieved.*

Sixty-eight percent of the strains were identified at species level and 12% at genus level. Those fungi that remained identified at higher rank were sterile in axenic culture and did not accomplished a univocal molecular result. Most of the taxa belonged to Ascomycota (91) while Basidiomycota (8) and Mucoromycota (3) were scarcely represented (Table 3).

In general, 10 classes, 12 orders and 32 families were represented. With the exception of Leotiomycetes (3%), Ascomycota were homogenously distributed in three classes: Dothideomycetes (30%, mainly Pleosporales and Capnodiales – 18% and 12%, respectively), Sordariomycetes (29%, mainly Hypocreales and Microascales – 13% and 6%, respectively) and Eurotiomycetes (26%, mainly Eurotiales – 24%). As for Basidiomycota, the most represented classes were Agaricomycetes (4%), Tremellomycetes (1%), Ustilaginomycetes (1%) and Wallemiomycetes (1%). The single representative of Mucoromycota was *Cunninghamella bertholletiae* (Mucorales).

*Penicillium* and *Cladosporium* were the most abundant genera with 17 and 9 species, and were isolated from all sites and plant parts, regardless of the culture conditions used.

**FIGURE 3:**
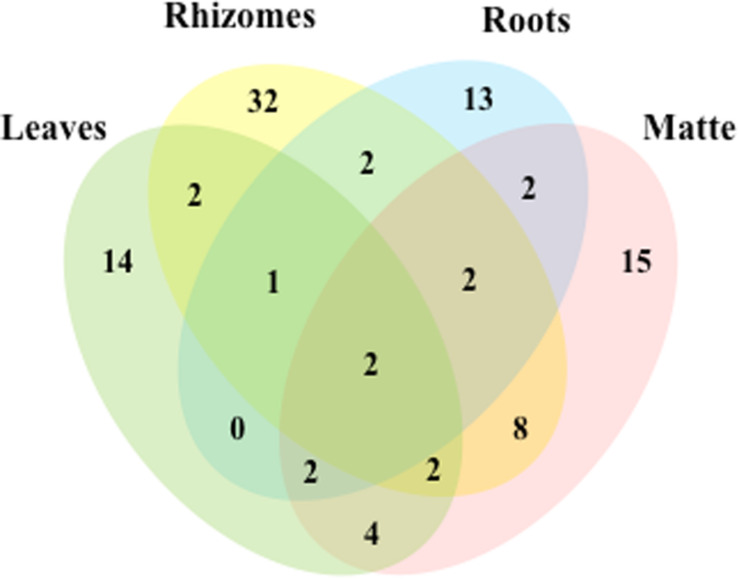
Venn diagram showing the total number of taxa and shared taxa among leaves, rhizomes, roots and matteof *P. oceanica*.

Out of the 102 taxa isolated, only *Cladosporium cladosporioides* and *Penicillium commune* were detected in all four plant parts; 7 and 18 taxa were isolated from three and two parts, respectively. The rest was exclusive to rhizomes (32), matte (15), leaves (14), and roots (13) (Figure 3).

**FIGURE 4:**
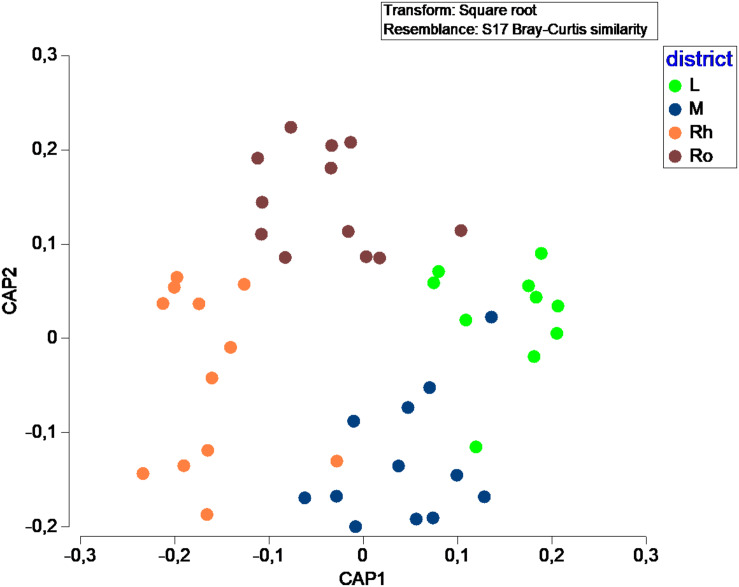
Canonical Analysis of Principal coordinates (CAP) illustrating the diversity of fungal communities among plant parts.

In terms of fungal species diversity, plant parts were significantly different (PERMANOVA; p ≤ 0.05; Figure 4), with the exception of leaves *vs* matte (PERMANOVA; p = 0.06). The most frequent species were *Penicillium chrysogenum* (72.5%) in leaves, *Corollospora* sp. (39.8%), *Corollospora maritima* (21.0%) and *Penicillium chrysogenum* (9.25%) in matte, *Penicillium antarcticum* (28.1%), *Lulworthiales* sp. (24.1%), *Aspergillus sydowii* (14.5%) and Microascales sp. (14.1%) in rhizomes, and finally *Lulworthiales* sp. (59.0%) and *Penicillium antarcticum* (23.8%) in roots (SIMPER analysis). No significant difference (PERMANOVA; *p* = 0.471) was observed between sampling sites.

**FIGURE 5:**
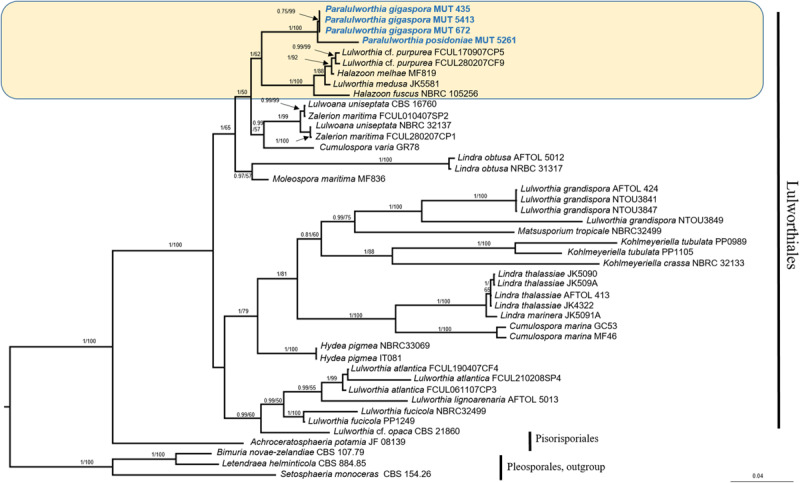
Bayesian phylogram of Lulworthiales based on a combined *nrSSU*, *nrITS*, and *nrLSU* dataset. The tree is rooted to Pleosporales. Branch numbers indicate BYPP/MLBvalues; Bar = expected changes per site (0.04).

The mycobiota of *P. oceanica* included four strains of Lulworthiaceae (MUT 435, MUT 672, MUT 5413, and MUT 5261) that are representatives of a novel genus consisting of two species. The phylogenetic tree based on *nrITS*, *nrSSU*, and *nrLSU* (Figure 5), together with the morphological inspection confirmed the novelty of *Paralulworthia gigaspora* sp. nov. and of *Paralulworthia posidoniae* sp. nov.

### Phylogenetic Inference and Taxonomy of *Paralulworthia gigaspora* and *Paralulworthia posidoniae*

Preliminary analyses carried out individually with *nrITS*, *nrSSU*, and *nrLSU* denoted no incongruence in the topology of the trees. The combined dataset, built on the basis of BLASTn results and of recent phylogenetic studies (Azevedo et al., 2017), consisted of 45 taxa (including MUT isolates) that represented 15 genera and 27 species. Twelve sequences (4 *nrITS*, 4 *nrSSU*, and 4 *nrLSU*) were newly generated, while 94 were retrieved from GenBank.

The combined dataset had an aligned length of 2135 characters, of which 1096 were constant, 352 were parsimony-uninformative and 687 parsimony informative.

The strains under investigation formed a strongly supported and separated clade (BYPP = 1.00; MLB = 100%) within Lulworthiaceae, unique family of the order Lulworthiales (Figure 5). Since this clade was close to the genus *Lulworthia sensu latu*, we introduced the novel genus *Paralulworthia* that includeed the two species *Paralulworthia gigaspora* and *Paralulworthia posidoniae*. The development of the sexual morphotype of MUT 435 and MUT 5261 on maritime pine wood confirmed the divergence between the two species.

*Paralulworthia* gen. nov.

MB 833285.

Type species: *Paralulworthia gigaspora* sp. nov.

Etymology: in reference to the phylogenetic closeness to the genus *Lulworthia sensu latu*.

Phylogenetic placement: Lulworthiaceae, Lulworthiales, Sordariomycetes, Ascomycota. The genus *Paralulworthia* gen. nov. clustered together with the genera *Lulworthia sensu latu* and *Halazoon* (Figure 5).

Description: growing efficiently on wood. *Ascomata* subglobose to ellipsoidal, single, scattered, immersed in the cortex or erumpent, brown to black at maturity; *peridium* dark brown composed of several layers of thick–walled cells of *textura angularis*. *Asci* containing 8 spores, dehiscent. Sterile elements not observed. Ascospores uni- to 3-septate at maturity, constricted at septa (particularly the central one), ellipsoid to fusiform, pointed at both ends, thin-walled, from light to dark brown at maturity, straight.

*Paralulworthia gigaspora* sp. nov.

**FIGURE 6:**
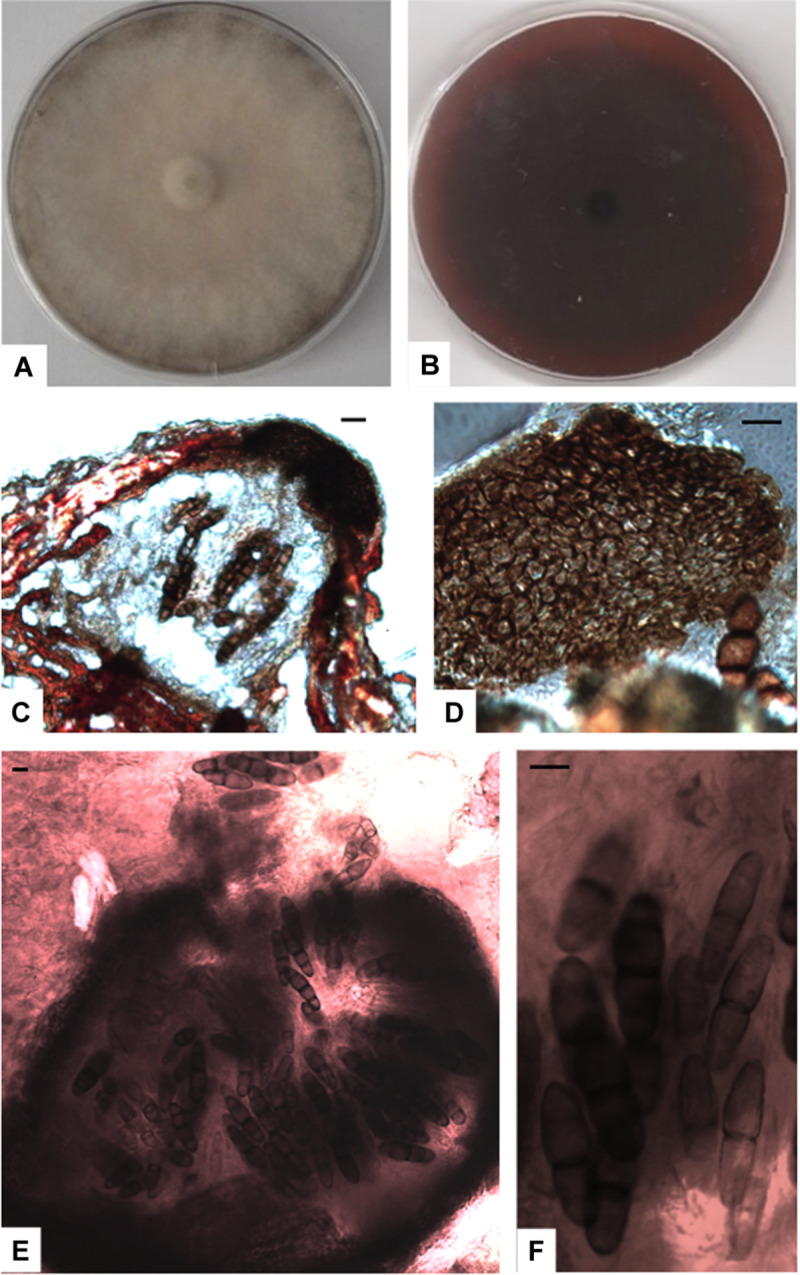
*Paralulworthia gigaspora* sp.nov. 28-days-old colony incubated at 21°C on MEASW **(A)** and reverse **(B)**; ascomata **(C,E)**, peridium with textura angularis **(D)**; asci with eight ascospores at different degrees of maturity **(F)**. Scale bars 20 μm.

MB 833288 (Figure 6).

Type: Europe, Italy, Tuscany, Mediterranean Sea, Elba Island (LI), Ghiaie ISL, 3–5 m depth, 42°49′04”N, 10°19′20″E March 2010, R. Mussat-Sartor and N. Nurra, on the sea grass *P. oceanica* rhizomes, MUT 435 holotype, living culture permanently preserved in metabolically inactively state by deep-freezing at MUT. MUT 672 and MUT 5413 = paratypes.

Etymology: in reference to the large size of the ascospores.

Description: growing actively on *P. pinaster*. *Hyphae* 3.2 μm wide, septate, from brownish to hyaline. *Chlamydospores* numerous, solitary and multicellular, from one to four celled, 2 × 3 μm diam., from light to dark brown, globose and subglobose.

Sexual morph: *Ascomata* 480-540 × 160–270 μm ($\bar{x}$ = 506.7 × 205 μm, *n* = 10), subglobose to ellipsoidal, single, scattered, immersed in the cortex or erumpent, brown to black at maturity; *peridium* dark brown 73-88 μm thick composed of several layers of thick–walled cells of *textura angularis*. *Asci* 115–130 × 30–40 μm ($\bar{x}$ = 118 × 31.8 μm, *n* = 10), fusiform to cylindrical, containing 8 spores, dehiscent, hyaline, vertically oriented to the host surface. Sterile elements not observed. *Ascospores* 76-83 × 19-25 μm ($\bar{x}$ = 79.3 × 21.7 μm, *n* = 10), uni- to 3-septate at maturity, constricted at septa (particularly the central one), ellipsoid to fusiform, pointed at both ends, thin-walled, from light to dark brown at maturity, straight.

Asexual morph not observed.

Colony description: growing on MEASW from 60 to 90 mm Ø after 28 days at 21°C, mycelium from feltrose to floccose, from whitish to ochre, reverse from light brown to bordeaux. A caramel colored diffusible pigment and exudates were often present.

*Paralulworthia posidoniae* sp. nov.

**FIGURE 7:**
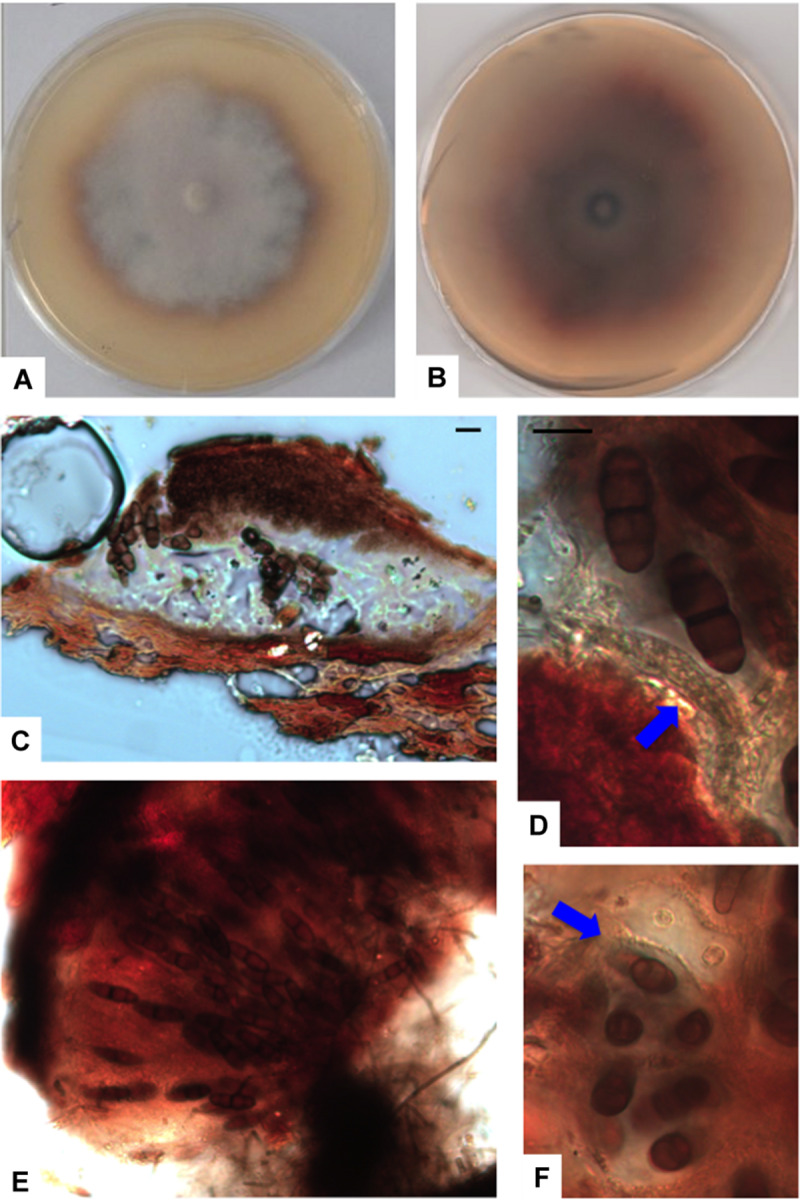
*Paralulworthia posidoniae* sp.nov. 28-days-old colony incubated at 21°C on MEASW **(A)** and reverse **(B)**; ascoma **(C)**, clavate young ascus (**D**, red arrow); mature asci in the ascoma **(E)**, transversal section of asci with ascospores (**F**, red arrow). Scale bars 20 μm **(C,E,F)**, 10 μm **(D)**.

MB 833291 (Figure 7).

Type: Europe, Italy, Tuscany, Mediterranean Sea, Elba Island (LI), Ghiaie ISL, 3–5 m depth, 42°49′04″N, 10°19′20″E March 2010, R. Mussat-Sartor and N. Nurra, on the sea grass *P.oceanica* rhizomes, MUT 5261 holotype, living culture permanently preserved in metabolically inactively state by deep-freezing at MUT.

Etymology: in reference to the original substrate, the seagrass *P. oceanica*.

Description: growing actively on *P. pinaster*. *Hyphae* 2.5–3.5 μm ($\bar{x}$ = 2.9 μm, *n* = 10) wide, septate, from hyaline to dematiaceous.

Sexual morph: *Ascomata* 365–450 × 155–230 μm ($\bar{x}$ = 405 × 191.3 μm, *n* = 10), ellipsoidal, single, scattered, immersed in the cortex or erumpent, brown to black at maturity; *peridium* dark brown 23-50 μm thick composed of several layers of thick-walled cells of *textura angularis*. *Asci* 150–190 × 40–60 μm ($\bar{x}$ = 171.3 × 52 μm, *n* = 10), containing 8 spores, clavate and early dehiscent. Sterile elements not observed. *Ascospores* oblong, 32–43 × 12–16 μm ($\bar{x}$ = 38.4 × 13.2 μm, *n* = 10), uni- to 3-septate at maturity, constricted at septa (particularly the central one), ellipsoid to fusiform, pointed at both ends, thin-walled, from light to dark brown at maturity, straight.

Asexual morph not observed.

Colony description: growing on MEASW, reaching 42 mm Ø after 28 days at 21°C, mycelium feltrose with irregular edges, whitish with slight shades of purple in the middle, gray-green at the edges; reverse caramel. A caramel colored diffusible pigment and exudates were often present.

## Discussion

Broadening the knowledge of marine fungi associated with a vast range of biotic and abiotic substrates is becoming more and more important. The reason behind this lies in the ecological relevance that these organisms may play in the oceans and in their possible exploitation as a source of potentially novel enzymes and bioactive compounds (Bovio et al., 2019; Grossart et al., 2019; Overy et al., 2019; Youssef et al., 2019).

Nowadays, *P. oceanica* is rapidly disappearing from the Mediterranean Sea, being seriously threatened by a number of factors^7^. Its mycobiota was partially studied in the past (Cuomo et al., 1985; Panno et al., 2013). Recently Vohnik and collaborators described the endophytes colonizing the roots of this endemic seagrass (Vohnik et al., 2017, 2019). Here, we investigated the cultivable mycobiota of *P. oceanica* with the intent of comparing it with the fungal communities of two photobionts of the same phytocoenosis.

### Mycobiota *of P. oceanica*

The observed total fungal load ranged from 5 × 10^2^ to 1.4 × 10^4^ CFU g^–1^ dw, approximately one order of magnitude higher than what reported by Panno et al. (2013) (up to 1.6 × 10^3^ CFU g^–1^ dw). Since the two sampling occurred in the same period of the year (March), we hypothesize that the climate factor did not affect the total load. The fungal abundance through the plant parts was also slightly different: in the present study, the fungal load followed the distribution rhizomes > leaves > matte > roots, whereas in the previous investigation matte and leaves were swapped (Panno et al., 2013). The use of specific culture media and of two incubation temperatures maximized the number of taxa isolated. Indeed, a lower temperature (15°C) allowed the growth of psychrotolerant/psychrophilic fungi such as four species of *Penicillium* (*P. canescens*, *P. glabrum*, *P. paneum*, *P. vinaceum*) (Table 2). Most probably, this is one of the reasons why we retrieved a number of taxa higher than what reported in 2013 by Panno (102 vs. 88).

**TABLE 2:** Dataset used for phylogenetic analysis.

**Species**	**Strain**	**Source**	**nrITS**	**nrSSU**	**nrLSU**
*Achroceratosphaeria potamia* Réblová, Fourn. & Hyde	JF 08139	Submerged wood of *Platanus* sp.	–	GQ996541	GQ996538
*Bimuria novae-zelandiae* Hawksw., Chea & Sheridan	CBS 107.79	Soil	–	AY016338	AY016356
*Cumulospora marina* Schmidt	MF46	Submerged wood	–	GU252136	GU252135
	GC53	Submerged wood	–	GU256625	GU256626
*C. varia* Chatmala & Somrithipol	GR78	Submerged wood	–	EU848593	EU848578
*Halazoon mehlae* Abdel-Aziz, Abdel-Wahab & Nagah.	MF819	Drift stems of *Phragmites australis*	–	GU252144	GU252143
*H. fuscus* (Schmidt) Abdel-Wahab, Pang, Nagah., Abdel-Aziz & Jones	NBRC 105256	Driftwood	–	GU252148	GU252147
*Hydea pigmaea* (Kohlm.) Pang & Jones	NBRC 33069	Driftwood	–	GU252134	GU252133
	IT081	Driftwood	–	GU256632	GU256633
*Kohlmeyeriella crassa* (Nakagiri) Kohlm., Volkm.-Kohlm., Campb., Spatafora & Gräfenhan	NBRC 32133	Sea foam	LC146741	AY879005	LC146742
*K. tubulata* (Kohlm.) Jones, Johnson & Moss	PP115	Marine environment	–	AY878998	AF491265
	PP0989	Marine environment	–	AY878997	AF491264
*Letendraea helminthicola* (Berk. & Broome) Weese ex Petch	CBS 884.85	Yerba mate	EU715680	AY016345	AY016362
*Lindra thalassiae* Orpurt, Meyers, Boral & Simms	JK 509A	Marine environment	–	U46874	U46891
	AFTOL 413	Marine environment	DQ491508	DQ470994	DQ470947
	JK 5090	Marine environment	–	AF195634	AF195635
	JK 4322	*Thalassia testudinum* leaves	–	AF195632	AF195633
*L. obtusa* Nakagiri & Tubaki	NRBC 31317	Sea foam	LC146744	AY879002	AY878960
	AFTOL 5012	Marine environment	–	FJ176847	FJ176902
*L. marinera* Meyers	JK 5091A	Marine environment	–	AY879000	AY878958
*Lulworthia lignoarenaria* (Koch & Jones) Kohlm., Volkm.-Kohlm., Campb., Spatafora & Gräfenhan	AFTOL 5013	Marine environment	–	FJ176848	FJ176903
*L. fucicola* Sutherl.	ATCC 64288	Intertidal wood	–	AY879007	AY878965
	PP1249	Marine environment	–	AY879008	AY878966
*L. grandispora* Meyers	AFTOL 424	Dead *Rhizophora* sp. branch	–	DQ522855	DQ522856
	NTOU3841	Driftwood	–	KY026044	KY026048
	NTOU3847	Decayed mangrove wood	–	KY026046	KY026049
	NTOU3849	Decayed mangrove wood	–	KY026047	KY026050
*L. medusa* (Ellis & Everh.) Cribb & Cribb	JK 5581	Spartina	–	AF195636	AF195637
*L. cf. purpurea* (Wilson) Johnson	FCUL170907CP5	Sea water	KT347219	KT347201	JN886824
	FCUL280207CF9	Sea water	KT347218	KT347202	JN886808
*L. opaca* (Linder) Cribb & J.W. Cribb	CBS 21860	Driftwood in seawater	–	AY879003	AY87896
*L. atlantica* Azevedo, Caeiro & Barata	FCUL210208SP4	Sea water	KT347205	KT347193	JN886843
	FCUL190407CF4	Sea water	KT347207	KT347198	JN886816
	FCUL061107CP3	Sea water	KT347208	KT347196	JN886825
*Matsusporium tropicale* (Kohlm.) Jones & Pang	NBRC 32499	Submerged wood	–	GU252142	GU252141
*Moleospora maritima* Abdel-Wahab, Abdel-Aziz & Nagah.	MF836	Drift stems of *Phragmites australis*	–	GU252138	GU252137
*Paralulworthia gigaspora* sp. nov.	MUT 435	*P. oceanica* – rhizomes	MN649242	MN649246	MN649250
	MUT 672	*P. oceanica* – rhizomes	MN649244	MN649248	MN649252
	MUT 5413	*P. oceanica* – rhizomes	MN649243	MN649247	MN649251
*Paralulworthia posidoniae* sp. nov.	MUT 5261	*P. oceanica* – rhizomes	MN649245	MN649249	MN649253
*Zalerion maritima* (Linder) Anastasiou	FCUL280207CP1	Sea water	KT347216	KT347203	JN886806
	FCUL010407SP2	Sea water	KT347217	KT347204	JN886805
*Lulwoana uniseptata* (Nakagiri) Kohlmeyer et al.	NBRC 32137	Submerged wood	LC146746	LC146746	LC146746
	CBS 16760	Driftwood	–	AY879034	AY878991
*Setosphaeria monoceras* Alcorn	CBS 154.26	n.d.	DQ337380	DQ238603	AY016368

*Genbank sequences include newly generated nrITS, nrLSU and nrSSU amplicons relative to the novel species Paralulworthia gigaspora and P. posidoniae.*

**TABLE 3:** Fungal taxa isolated from *Posidonia oceanica*.

**Taxa**	**From Mediterranean Sea**	**From *P. oceanica***
*Acrodontium pigmentosum* Videira & Crous*	FR	FR
*Acrostalagmus luteoalbus* (Link) Zare, W. Gams & Schroers	[1], [2]	FR
*Alfaria dandenongensis* Crous*	FR	FR
*Alternaria* sp.	–	–
*Arthrinium arundinis* (Corda) Dyko & B. Sutton	[1], [2], [3]	[3]
*Arthrinium* sp.	–	–
*Aspergillus conicus* Blochwitz	[4]	FR
*Aspergillus fumigatus* Fresen.	[2], [3]	[3]
*Aspergillus pseudoglaucus* Blochwitz*	FR	FR
*Aspergillus sydowii* (Bainier & Sartory) Thom & Church*	[2], [5]	FR
*Aspergillus versicolor* (Vuill.) Tirab.*	[2], [3]	[3]
*Aureobasidium pullulans* (De Bary) G. Arnaud ex Cif., Ribaldi & Corte	[4]	FR
*Beauveria bassiana* (Bals.-Criv.) Vuill.	[1]	[3]
*Bjerkandera adusta* (Willd.) P. Karst.	[4]	FR
*Cadophora* sp.	–	–
*Capnodium* sp.	–	–
*Cerrena unicolor* (Bull.) Murrill*	FR	FR
*Chrysosporium lobatum* Scharapov*	FR	FR
*Chrysosporium undulatum* P. Vidal, Guarro & Ulfig*	FR	FR
*Cladosporium aggregatocicatricatum* Bensch, Crous & U. Braun*	FR	FR
*Cladosporium allicinum* (Fr.: Fr.) Bensch, U. Braun & Crous	[1], [2], [4]	FR
*Cladosporium cladosporioides* (Fresen.) G.A. de Vries	[1], [2], [3], [6], [7]	[3]
*Cladosporium halotolerans* Zalar, de Hoog & Gunde-Cim.	[2], [6]	FR
*Cladosporium herbarum* (Pers.) Link	[1], [3]	[3]
*Cladosporium pseudocladosporioides* Bensch, Crous & U. Braun	[2], [4], [6], [7]	FR
*Cladosporium sphaerospermum* Penz.	[1], [4]	[3]
*Cladosporium velox* Zalar, de Hoog & Gunde-Cim.*	FR	FR
*Cladosporium xylophilum* Bensch, Shabunin, Crous & U. Braun	[4]	FR
*Cordyceps farinosa* (Holmsk.) Kepler, B. Shrestha & Spatafora*	FR	FR
*Corollospora anglusa* Abdel-Wahab & Nagah.*	[8]	FR
*Corollospora maritima* Werderm.*	[6], [8], [9], [10], [11], [12], [13], [14], [15],	[9], [14]
*Corollospora* sp.	–	–
*Cunninghamella bertholletiae* Stadel*	FR	FR
*Dasyscyphus virgineus* (Batsch) Gray*	FR	FR
*Didymella fabae* G.J. Jellis & Punith.*	FR	FR
*Dioszegia* sp.	–	–
*Elbamycella rosea* A. Poli, E. Bovio, V. Prigione & G.C. Varese	[16]	[16]
*Engyodontium album* (Limber) de Hoog*	[6], [17]	FR
*Fusarium venenatum* Nirenberg*	FR	FR
*Gibellulopsis nigrescens* (Pethybr.) Zare, W. Gams & Summerb.	[1], [2], [3], [4], [6]	[3]
*Halosarpheia japonica* Abdel-Wahab & Nagah.*	FR	FR
Hypocreales sp.	–	–
*Irpex lacteus* (Fr.) Fr.*	[16]	FR
*Lophiotrema rubi* (Fuckel) Y. Zhang ter, C.L. Schoch & K.D. Hyde	[1], [3]	[3]
Lulworthiales sp.	–	–
*Massarina* sp.	–	–
*Metarhizium carneum* (Duché & R. Heim) Kepler, S.A. Rehner & Humber*	FR	FR
Microascaceae sp.	–	–
Microascales sp.	–	–
*Microsphaeropsis arundinis* (S. Ahmad) B. Sutton*	[1], [2], [3]	[3]
*Neoroussoella solani* (Crous & M.J. Wingf.) Jayasiri & K.D. Hyde*	FR	FR
*Neoroussoella* sp.	–	–
*Nigrospora oryzae* (Berk. & Broome) Petch*	FR	FR
Onygenaceae sp.	–	–
*Ophiognomonia setacea* (Pers.) Sogonov*	FR	FR
*Paracamarosporium psoraleae* Crous & M.J. Wingf.*	FR	FR
*Paraconiothyrium variabile* Riccioni, Damm, Verkley & Crous	[4]	FR
*Paralulworthia gigaspora* sp. nov.*	FR	FR
*Paralulworthia posidoniae* sp. nov.*	FR	FR
*Paraphaeosphaeria michotii* (Westend.) O.E. Erikss.*	FR	FR
*Penicillium antarcticum* A.D. Hocking & C.F. McRae	[1], [2], [4]	FR
*Penicillium brevicompactum* Dierckx	[1], [2], [3], [4]	[3]
*Penicillium canescens* Sopp.*	[2]	FR
*Penicillium chrysogenum* Thom	[1], [2]	[3]
*Penicillium commune* Thom	[1]	FR
*Penicillium cremeogriseum* Chalab.*	FR	FR
*Penicillium crustosum* Thom	[1]	FR
*Penicillium cvjetkovicii* S. W. Peterson, Z. Jurjevic & J. C. Frisvad*	FR	FR
*Penicillium glabrum* (Wehmer) Westling*	FR	FR
*Penicillium janczewskii* K.M. Zalessky*	[3]	[3]
*Penicillium paneum* Frisvad*	FR	FR
*Penicillium pinophilum* Hedgc.*	FR	FR
*Penicillium roseopurpureum* Dierckx	[4]	FR
*Penicillium solitum* Westling	[1]	FR
*Penicillium* sp.	–	–
*Penicillium steckii* K.M. Zalessky	[4], [18]	FR
*Penicillium vinaceum* J.C. Gilman & E.V. Abbott*	[2]	FR
*Phoma* sp.	–	–
Plectosphaerellaceae sp.	–	–
Pleosporales sp.	–	–
*Pleurophoma* sp.	–	–
*Porostereum spadiceum* (Pers.) Hjortstam & Ryvarden	FR	FR
*Preussia funiculata* (Preuss) Fuckel*	FR	FR
*Preussia terricola* Cain*	FR	FR
*Pseudozyma prolifica* Bandoni	FR	FR
*Purpureocillium lilacinum* (Thom) Luangsa-ard, Houbraken, Hywel-Jones & Samson*	[6]	FR
*Pyrenochaeta* sp.	–	–
*Pyrenochaetopsis leptospora* (Sacc. & Briard) Gruyter, Aveskamp & Verkley*	FR	FR
*Ramularia* sp.	–	–
*Rhexocercosporidium carotae* (Årsvoll) U. Braun	[1], [19]	[19]
Roussoellaceae sp.	–	–
*Sarocladium bacillisporum* (Onions & G.L. Barron) Summerb.*	FR	FR
*Scedosporium apiospermum* (Sacc.) Sacc. ex Castell. & Chalm.*	[6]	FR
*Schizophyllum commune* Fr.	[1], [3], [4]	[3]
Sordariomycetes sp.	–	–
*Talaromyces assiutensis* Samson & Abdel-Fattah*	FR	FR
*Talaromyces trachyspermus* (Shear) Stolk & Samson*	FR	FR
*Tolypocladium cylindrosporum* W. Gams*	FR	FR
*Trichoderma atroviride* P. Karst.*	[6]	FR
*Trichoderma harzianum* Rifai	[2], [3], [4], [18]	[3]
*Wallemia sebi* (Fr.) Arx	[3], [4]	[3]
*Zopfiella ebriosa* Guarro, P.F. Cannon & Aa*	FR	FR

FR, First Record from the Mediterranean Sea and/or P. oceanica. *Species isolated only from P. oceanica within the phytocoenosis. References: [1] Gnavi et al. (2017); [2] Bovio et al. (2017); [3] Panno et al. (2013); [4] Garzoli et al. (2018); [5] Greco et al. (2017); [6] Garzoli et al. (2015); [7] Garzoli et al. (2014); [8] Abdel-Wahab et al. (2009); [9] Vohník et al. (2016); [10] Jones et al. (1972); [11] Montemartini (1979); [12] Furtado and Jones (1980); [13] Grasso et al. (1985); [14] Cuomo et al. (1988); [15] Grasso et al. (1990); [16] Poli et al. (2018); [17] Proksch et al. (2010); [18] Paz et al. (2010); [19] Gnavi et al. (2014).

As expected, Ascomycota prevailed, confirming once again the dominance of this phylum in the oceans^8^. In detail, *Penicillium* and *Cladosporium*, were widely represented in all plant parts and sampling sites, and were characterized by the highest number of species (17 and 9, respectively). Members of *Penicillium* and *Cladosporium* are commonly reported in marine environments worldwide, probably due to the high adaptation to the peculiar chemical-physical conditions (Raghukumar, 2017). On the other side, it is not unreasonable to relate the high abundance of these genera to their extraordinary sporulation rate that may end in an overestimation of the substrate colonization (Imhoff et al., 2011).

Interestingly, 37 species were retrieved for the first time from the Mediterranean Sea and 61 from *P. oceanica* (Table 2), while only 19 species were previously detected in *P. oceanica* (Cuomo et al., 1985; Panno et al., 2013; Gnavi et al., 2014). Several species, besides being isolated for the first time from the Mediterranean Sea and from *P. oceanica*, were never found in any marine habitat before. For instance, this is the case of *Alfaria dandenongensis*, *Aspergillus pseudoglaucus*, *Cerrena unicolor*, *Didymella fabae*, and *Neoroussoella solani*. *A. pseudoglaucus* is an opportunistic human pathogen (Siqueira et al., 2018) as well as *N. solani*, a causal agent of keratomycosis (Mochizuki et al., 2017), while their underwater ecological role is still obscure. On the contrary, we can hypothesize the role of the plant pathogen *D. fabae* and of the leaf litter saprobe *A. dandenongensis*^9^. Similarly, it is possible to assume an active role for white rot fungi (i.e., *Bjerkandera adusta*, *C. unicolor* and *Schizophyllum commune*) in transforming recalcitrant matrixes (Poli et al., 2018). Finally, entomopathogenic fungi like *Cordyceps farinosa*, due to their ability to degrade chitin, may be capable of attacking the shellfish carapace.

### Distribution Among Plant Parts

The mycobiota of *P. oceanica* changed significantly among the four parts of the plant, with the exception of leaves vs. matte. This last observation could be ascribable to the similar structure of the substrates: the main components of matte are the decaying and fluctuating leaves (Vohnik et al., 2017). The presence of antagonistic organisms, both micro- and macro-, together with the specific localization of toxic metabolites, such as tannic acid in the leaves (Pergent et al., 2008), may affect the distribution among plant parts. It is not unexpected to retrieve unique mycobiota on differently organized and composed parts of a complex plant, as occurs in terrestrial phanerogams.

The only two species common to the four regions were *C. cladosporioides* and *P. commune*: the former is a usual inhabitant of the sea (Garzoli et al., 2015), the latter, although observed for the first time on *P. oceanica*, was already found associated with *F. petiolata* (Gnavi et al., 2017). *P. chrysogenum* dominated the mycobiota of the leaves, whereas the marine genus *Corollospora* prevailed in matte. With respect to rhizomes and roots, two taxa (Lulworthiales sp. and *P. antarcticum*) were the main components of the fungal communities, even though their proportions were different in the two plant parts. Vohník et al. (2016) found members of the order Lulworthiales in the rhizomes and roots of *P. oceanica.* Lulworthiales degrade wood and marsh plants in marine and estuarine environments and seem to be the most common seagrasses decomposers followed by *Corollospora maritima*. Indeed, *C. maritima* and *Lulworthiales* spp., are well-known cellulases producers and can break down complex ligno-cellulose compounds, making them available for other organisms nourishment (Raghukumar, 2017). Interestingly, two new species of Lulworthiales were found in rhizomes and described here for the first time.

The retrieval of *P. chrysogenum* in leaves and, to a lower extent in matte, may be related to defense mechanisms against predators and/or pathogens, as demonstrated in terrestrial plants, where the presence of this fungus stimulates the synthesis of secondary metabolites such as salycilic and jasmonic acids (Chen et al., 2018).

### The Mycobiota of *P. oceanica* Compared With the Fungal Communities of *F. petiolata* and *P. pavonica*

*F. petiolata* and *P. pavonica* are two algae that characterize the Neptune forests. Since their mycobiota were recently described (Gnavi et al., 2017; Garzoli et al., 2018), we considered worthwhile to compare the fungal communities associated with these three organisms that grow only few centimeters apart. To this end, one must bear in mind that samplings occurred at the same time and in the same sites (Ghiaie and Margidore): it would be interesting to clarify whether a strict specificity between fungi and photobionts exists.

**FIGURE 8:**
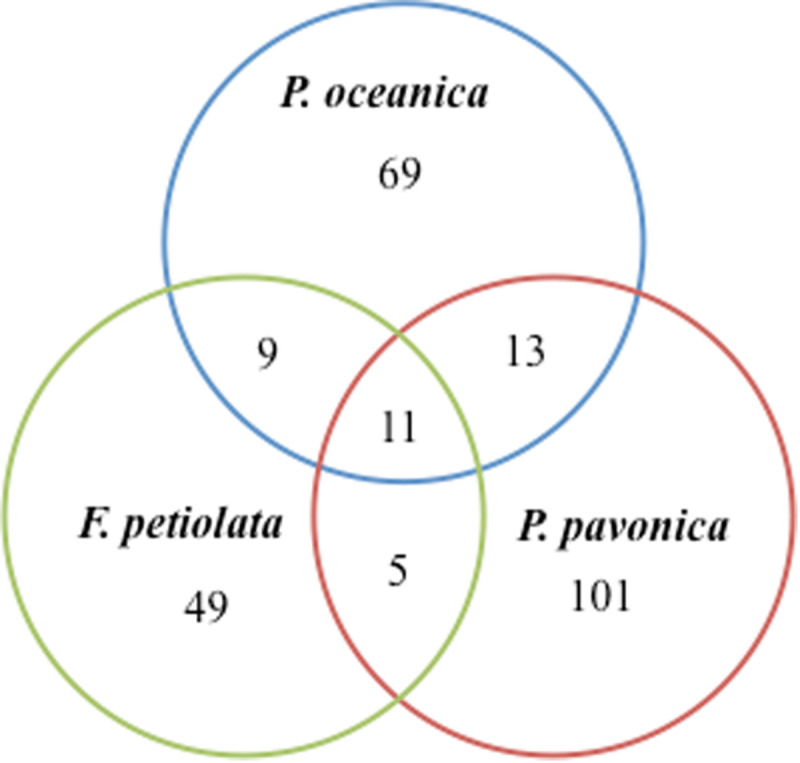
Venn diagram showing the total number of taxa and shared taxa among the three organisms.

**FIGURE 9:**
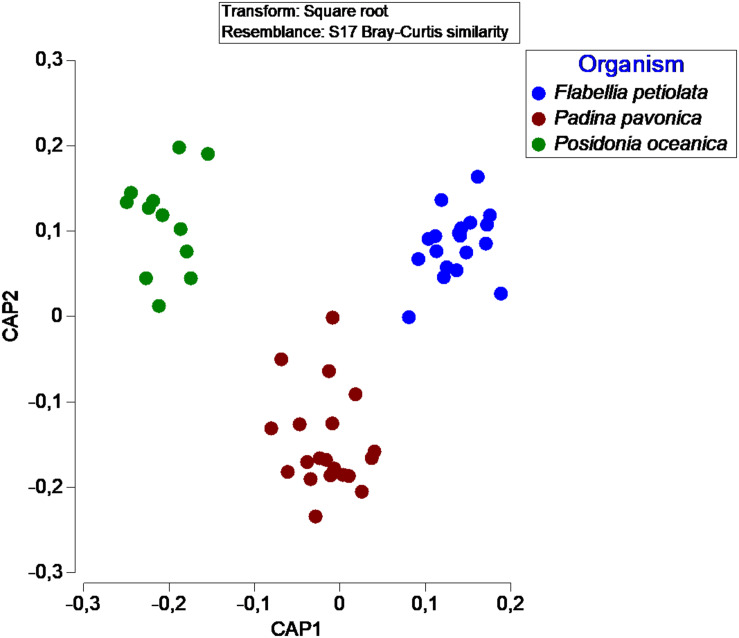
Canonical Analysis of Principal coordinates (CAP) illustrating the diversity of fungal communities associated with different organisms.

In terms of number of taxa, *P. pavonica* displayed the richest mycoflora, followed by *P. oceanica* and finally by *F. petiolata*, where the number of taxa was almost halved. As shown in Figure 8, *P. oceanica* and the two algae had 11 species in common: *Acrostalagmus luteoalbus*, *Arthrinium arundinis*, *Cladosporium allicinum*, *C. cladosporioides*, *C. sphaerospermum*, *Gibellulopsis nigrescens*, *Massarina rubi*, *P. antarcticum*, *P. brevicompactum*, *P. roseopurpureum*, and *S. commune*. Besides their broad diffusion in the oceans, these fungi, due to the secretion of secondary metabolites (e.g., antialgal, antifungal, and/or antibacterial) may help in deterring colonization of their hosts by other microbes, and in warding off predators (Raghukumar, 2017). On the other side, the three substrates show specific mycobiota, significantly different from each other (Figure 9), with high and equivalent percentages of dissimilarity (94.68–95.74%). The uniqueness of each community derives from the prevalence of exclusive taxa (Figure 8).

Confirming the “substrate specificity” is also the fact that there is a significant difference between the mycobiota of the three organisms even if the “non-photosynthesizing” parts of *P. oceanica* were excluded from the analysis. The leaves of *P. oceanica* are in fact characterized by a mycobiota significantly different from that of the other two photobionts (data not shown).

This divergence in “substrate specificity” may be due to the different composition and morphology of the hosts (e.g., *P. pavonica* with its hairy surface could entrap a high number of fungal propagules, contrary to the slippery texture of *F. petiolata*) and/or to the different metabolites produced by seagrasses and algae. Extracts of *P. oceanica* were proved active against bacteria, dermatophytes, and yeasts (Bernard and Pesando, 1989) as well as against *Trypanosoma brucei rhodesiense* and *Leishmania donovani* (Orhan et al., 2006). Likewise, the activity of *P. pavonica* extracts against bacteria and fungi was comparable to the efficiency showed by antibiotics and antimycotics used as control (Al-Enazi et al., 2018). Antibacterial, antiviral, antimitotic and antifungal properties were also detected in raw extracts of *F. petiolata* (Ballesteros et al., 1992). However, whether the macro-organisms, their microbiota, or the holobiont derived from their strict interaction is responsible for the production of the active compounds is still unclear. With the exclusion of cosmopolitan and/or widespread fungi, we can hypothesize the shaping of a relation between the microorganisms and their host, as recently demonstrated for three Atlantic sponges (Bovio et al., 2018). The establishment of a holobiont depends on both active (selection) and passive (drift) events. Due to the high connectivity of aquatic environments, differences in microbiota may be dependent on a combination of selection and drift. In most cases, fungi spread by passive dispersion of hyphal fragments and/or propagules that happen to adhere to the host. Their further development is consequent to the compatibility and interdependence between the individual organisms. In other words, the holobiont is an organism with its own features, modeled upon a mutual selection. The unimportance of the sampling site supports this idea. Ghiaie and Margidore are two extremely different sites both geographically (Ligurian Sea and Tyrrhenian Sea, respectively) and for the anthropic influence: while the former is located in a marine protected area, the latter is subjected to an intense anthropic disturbance. Nevertheless, no significant difference was detected in terms of fungal biodiversity.

An often-underappreciated ecological function of holobionts is their ability to restrain and nourish rare species: the host provides a niche favorable for the growth of specific communities different from those of the surrounding environment (Dittami et al., 2019). To this regard, we may define “rare” the two novel species here introduced.

### *Paralulworthia gigaspora* and *P. posidoniae*

This investigation introduces two new species that belong to a novel genus of Lulworthiaceae, the sole family of the order Lulworthiales (Sordariomycetes). Lulworthiaceae spp. are strictly marine species found on a range of substrates (e.g., submerged wood, seaweed, seafoam, etc.). This group includes the following genera: *Cumulospora*, *Halazoon*, *Hydea*, *Kohlmeyerella*, *Lulwoana, Lulworthia*, *Lindra*, *Matsusporium*, and *Moleospora*. The new genus *Paralulworthia* accommodates the four strains MUT 435, MUT 672, MUT 5413, and MUT 5261. *P. gigaspora* and *P. posidoniae* formed a strongly supported cluster segregated from the genus *Lulworthia sensu latu*. The previously suggested polyphyletic nature of *Lulworthia* spp. (Azevedo et al., 2017), that discriminate *Lulworthia sensu latu* from *Lulworthia* s*ensu strictu* is here confirmed and justifies the introduction of the new genus *Paralulworthia*. Besides, members of the genus *Lulworthia* are morphologically characterized by filamentous hyaline ascospores (Kohlmeyer et al., 2000), well distinct from the ellipsoidal brown ascospores of the new genus. Furthermore, beyond the phylogenetic distance, the dimension of the ascomata and ascospores allowed the recognition of two species. *P. gigaspora* and *P. posidoniae* were isolated exclusively from rhizomes that are charachterized by a high content of lignin (Kaal et al., 2016). Reproductive structures were detected only following colonization of *P. pinaster* wood specimens submerged in sea-water. These two observations indicate a possible lignicolous nature of these species.

## Conclusion

The seagrass *P. oceanica* is home to a huge variety of fungi. The comparison of our data with those previously obtained on two algae collected in the same phytocoenosis underlines a substrate-specificity. Besides, we demonstrated a plant-part-specificity of the fungal communities associated with the host. Marine phanerogames, likewise their terrestrial counterparts, enroll distinct microbiomes in different regions (i.e., phylloplane, rhizoplane etc.). This selective distribution seems to increment the resistance of plant to pathogens and predators by producing chemo-attractants and anti-microbial compounds. This is even more important for *Posidonia* meadows that are seriously threatened by climate changes, allochthonous species and anthropic activities. Nonetheless, further studies will need to clarify the mechanisms (e.g., molecular, physiological, etc.) at the basis of the specific interaction between microorganisms and hosts responsible for the shaping of a holobiont.

## Data Availability Statement

The sequences generated in this study can be found in GenBank. The accession numbers of the sequences deposited in GenBank are: MK578237–MK578251; MK581058–MK581072; MK626711–MK626722; MN165499–MN165504; MN173027–MN173033; MN177616–MN177627; MN173595–MN173610.

## Author Contributions

AP conducted molecular studies, phylogenesis, data analysis, and manuscript writing. EB and LR carried morphological analysis. VP was responsible for morphological analysis and manuscript writing. GV supervised the work.

## Conflict of Interest

The authors declare that the research was conducted in the absence of any commercial or financial relationships that could be construed as a potential conflict of interest.

## Footnotes

1 www.marinefungi.org

2 http://www.genecodes.com

3 www.mut.unito.it

4 http://trimal.cgenomics.org

5 http://tree.bio.ed.ac.uk/software/figtree

6 www.graphpad.com

7 www.eea.europa.eu/data-and-maps/figures/distribution-of-the-marine-angiosperm-posidonia-oceanica-and-zostera-sp-in-the-mediterranean

8 www.marinefungi.org

9 www.FUNGuild.org
